# 

**Published:** 1841-01

**Authors:** 


### CONTENTS

OF NO. I.

---

### ORIGINAL COMMUNICATIONS.

### ESSAYS AND CASES.

ART. I.—Memoir on the General Characters of Rachitis; read before the Royal Academy of Sciences of Paris, July, 1837. By JULIUS GUERIN, M. D. Translated from the French for the Western Journal by THOMAS W. COLESCOTT, M. D., of Cincinnati. - 1

### SELECTIONS FROM FOREIGN AND AMERICAN JOURNALS.

Cases of Poisoning by Arsenic. Treated by the Hydrated Peroxide of Iron. By T. T. SMILEY, M. D., and J. M. WALLACE, M. D. - - - - - - - - - - 55
Division of the Muscles of the Eye for Strabismus. By J. H. DIX, M. D., of Boston. - - - - - - - 59
Precocity in a Boy. - - - - - - - - - 61
On the Employment of Lactate of Iron. By MM. GELIS and CONTE. - - - - - - - - - - 62

Complete Absence of Menstruation. By M. Kruger-Hansen. 63
Colocynth Oil, a Substitute for Croton Oil in Neuralgia, Sciatica, &c. By M. G. Janelli. - - - - - - 63
Congestion of the Kidneys. - - - - - - - 64
Danish Medical Statistics. - - - - - - - 64

ORIGINAL INTELLIGENCE.

Hospitals of Paris. By M. L. Linton, M. D. - - - 65
Rachitis. - - - - - - - - - - 74
The American Me ical Almanac, for 1841. - - - - 75
Stokes and Bell's Practice. - - - - - - 79
Medical Institute of Louisville. - - - - - - 80

